# Effect of New Zealand blackcurrant extract on rest and walking-induced cardiorespiratory and metabolic responses in young adult Southeast Asian women

**DOI:** 10.1007/s00421-026-06215-8

**Published:** 2026-04-03

**Authors:** Mark E.T. Willems, Sorrawit Wannasorn, Kawissara Leelitthum, Amornpan Ajjimaporn, Alisa Nana

**Affiliations:** 1 School of Sport, Science and Engineering, University of Chichester, College Lane, Chichester, PO19 6PE UK; 2 College of Sports Science and Technology, Mahidol University, Salaya, Nakhon Pathom 73170 Thailand

**Keywords:** anthocyanins, women, substrate oxidation, walking, cardiovascular function, blackcurrant, exercise, ethnicity

## Abstract

Beneficial metabolic and cardiovascular effects of anthocyanin-rich New Zealand blackcurrant (NZBC) were primarily observed in studies with female and male Caucasian participants. We examined the effects of 14-day intake of NZBC extract (daily 210 mg of anthocyanins) during supine rest and moderate-intensity treadmill walking in Southeast Asian women (*n* = 16, age: 23 ± 5 years, BMI: 22.9 ± 3.8 kg·m^− 2^, body fat (BF): 35 ± 5%). A randomized, double-blind, placebo-controlled, cross-over design was used. Beat-by-beat (PhysioFlow^®^ Endure™) and breath-by-breath (Cortex Metalyzer 3B) measurements were recorded during 10-min of supine rest and 30-min of moderate-intensity treadmill walking (speed: 4.84 ± 1.00 km·h^− 1^). At rest, NZBC extract had no effect on the cardiovascular, physiological and metabolic responses. During moderate-intensity exercise, only lower systemic vascular resistance (15%) was observed in 50% of the participants based on the smallest worthwhile change analysis (*P* = 0.08 for the cohort with moderate effect size, *d* = 0.62). There was a significant correlation between fat oxidation during rest and moderate-intensity walking in the placebo and NZBC extract condition. There was a trend (*P* = 0.08) for the linear relationship between the walking-induced placebo respiratory exchange ratio and change in fat oxidation by intake of NZBC extract. In Southeast Asian women, 14-day anthocyanin-rich NZBC extract did not elicit consistent cardiorespiratory and metabolic effects, although some participants showed reduced systemic vascular resistance. It is possible that there was not the bioavailability of plasma anthocyanin-induced metabolites that could alter cell function for metabolic responses. Further research should optimize dosing strategies and examine the roles of ethnicity, sex, and anthocyanin-induced metabolite bioavailability in shaping responsiveness to the intake of anthocyanins.

## Introduction

The intake of dietary supplements by athletes is common (Garthe and Maughan 2018). Athletes consume dietary supplements with the aim to enhance physical training sessions, improve competitive exercise performance and support post-exercise recovery (for reviews see Peeling et al. 2019; Salem et al. 2024; Wang et al. 2024a, b). For the general population, however, dietary supplements are taken to potentially off-set nutritional deficiencies from habitual food intake (e.g. vitamins) but also with the intent to support the normal diet to provide health benefits (for reviews see Solnier et al. 2023; Wang et al. 2024a, b). Inter-individual responses for exercise and health effects by the intake of dietary supplements by athletes and the general population are normal (Margaritelis et al. 2023; Ramos-Romero et al. 2021). Factors such as biological sex (Yamaguchi et al. 2025), age (Capó et al. 2016) and training status (Tomáš Hlinský et al. 2020) contribute to this variability.

In the last decade, supplementation with anthocyanins by powder or extract made from New Zealand grown blackcurrant (Ribes nigrum L., family Grossulariaceae) has provided evidence for performance-enhancing effects [16.1 km time-trial (Cook et al. 2015) and high-intensity incremental treadmill running (Perkins et al. 2024)]. Health outcomes include improved insulin sensitivity (Willems et al. 2017), greater fat oxidation (Willems and Cook 2025), and reduction in central arterial stiffness and blood pressure (Okamoto et al. 2020). Anthocyanins (i.e. glycosylated anthocyanidins) are members of the flavonoid polyphenol family (Cheynier 2005) with dark-colored berries, fruits and vegetables rich sources of anthocyanins (He and Giustu 2010). In addition to the numerous studies on effects by intake of blackcurrant powder or extract, studies with anthocyanin-rich haskap berry (Lonicera caerulea L., family Caprifoliaceae) (Howatson et al. 2022), chokeberry (Vaccinium spp., family Ericaceae) (Cikiriz et al. 2021) and blueberry (Vaccinium spp., family Ericaceae) (Anders et al. 2021) have also provided beneficial exercise performance and metabolic effects making the general case for studies on the applied effects of anthocyanin-rich dietary supplements for sport, exercise and health. However, dark-colored berries and fruits, for example, can differ substantially with respect to anthocyanin composition (Wu et al. 2006). In the case of blackcurrant, it contains primarily (i.e. ~97–98% of anthocyanin composition) the anthocyanins delphinidin-3-rutinoside, delphinidin-3-glucoside, cyanidin-3-rutinoside and cyanidin-3-glucoside (Šimerdová et al. 2021). The beneficial exercise and health effects of anthocyanins are likely mediated through the anti-oxidant and anti-inflammatory effects of the anthocyanins and anthocyanin-induced metabolites.

The research on the effects of anthocyanin-rich powder and extract intake from New Zealand grown blackcurrants has shown alteration of metabolic and cardiovascular responses at rest and during exercise in studies with primarily Caucasian female and male participants. For example, during moderate-intensity walking, Willems et al. (2022) observed with an intake of 1-week of NZBC extract (210 mg anthocyanins per day) in Caucasian female participants an increase in fat oxidation with moderate-intensity treadmill walking that correlated with body mass index and body fat (%) in arms, legs and trunk. In addition, Şahin et al. (2021) observed in male Caucasian participants that 1-week intake of NZBC extract (210 mg anthocyanins per day) enhanced treadmill walking-induced fat oxidation by 11%. In addition, in the same cohort, there was also an increase in cardiac output and a reduced total peripheral resistance during moderate-intensity treadmill walking (Şahin et al. 2023). In contrast, in Willems et al. (2018), a study with matching methodology for dosing strategy and exercise modality and intensity as in Şahin et al. (2021), Southeast Asian men did not respond with a change in exercise-induced substrate utilization and only a trend for cardiovascular changes. This would suggest that ethnicity is another factor that needs to be considered for our understanding of individual responses to the intake of dietary supplements. Supporting this, Akins et al. (2021) demonstrated race-specific differences for the effect of dietary nitrate on the cardiovascular mental stress response. However, it remains possible that the absence of a metabolic response in the Southeast Asian men to the intake of anthocyanin-rich blackcurrant (Willems et al. 2018) was due to the dosing strategy, i.e. either a higher daily dose of anthocyanins or a longer intake duration would have been required to obtain a response. Dose and intake duration of NZBC extract can affect the responses. A dose-response study with 7 days intake of NZBC extract in Caucasian male cyclists showed different exercise-induced metabolic responses with 315 mg of anthocyanins than 105 mg of anthocyanins during 2 h of cycling at an intensity of 65% of maximum oxygen uptake (Cook et al. 2017). In addition, intake duration observations in Şahin et al. (2021, 2023) seem to suggest that metabolic and some cardiovascular responses with a two-week intake provided larger changes than 1-week intake during moderate-intensity walking. The majority of supplementation studies to examine exercise effects have focused on men, highlighting the need for more research involving female participant (Smith et al. 2022).

Therefore, the primary aim of the present study was to examine the effects of a two-week intake of anthocyanin-rich New Zealand blackcurrant extract on the cardiovascular, physiological and metabolic responses during supine rest and moderate-intensity walking in Southeast Asian females. In Caucasian men, resting fat oxidation correlated significantly with exercise-induced maximal fat oxidation (Robinson et al. 2016). In Southeast Asian men, there was a significant correlation between whole-body resting fat oxidation and exercise-induced fat oxidation (Willems et al. 2018). Such information is not available for Southeast Asian females. Therefore, the second aim of the study was to examine whether there is a significant correlation between whole-body fat oxidation at rest and during moderate intensity exercise in Southeast Asian women. Finally, the third aim was to examine the relationship between body fat percentage and the changes in exercise-induced fat oxidation in Southeast Asian women, as previously shown in Caucasian women (Willems et al. 2022).

## Methods

### Participants

Sixteen healthy Southeast Asian (i.e., Thai) women participated in the study (age: 23 ± 5 years, height: 160 ± 4 cm, body mass: 58.4 ± 11.4 kg, body mass index (BMI): 22.9 ± 3.8 kg·m^− 2^ (mean ± SD) (range BMI: 19.1–30.8, eleven with normal weight, three overweight and two obese (World Health Organisation 2004). The participants were recreationally active, engaging in habitual physical activity as part of daily living and leisure-time activities, but were not involved in structured physical training programs at the time of the study. This activity profile is consistent with participants in previous studies examining the physiological and metabolic effects of New Zealand blackcurrant extract in non-athletic populations. A cash honorarium was provided for participation in the study. Participants provided written informed consent after explanation of the aims of the study, the experimental procedures, the participant’s requirements for testing and potential risks and benefits. The participants were non-smokers, had no known allergies to anthocyanins and were not taking other dietary supplementation. Ethical approval for the study was obtained from Mahidol University (approval code: MU-CIRB 2022/091.2908, approval date: 29/08/2022). Protocols and procedures of the study adhered to the principles of the 2013 Declaration of Helsinki.

### Experimental design

The study had a double-blind, placebo-controlled, randomized cross-over design. Randomization was done with a standard coin flip. Nine participants were tested first in the placebo condition following the familiarization visit. Participants had three visits in the morning over a period of 2 months to the air-conditioned exercise physiology laboratory at the College of Sports Science and Technology at Mahidol University Thailand (temperature: 27.5 ± 0.9 °C, humidity: 44 ± 4%, barometric pressure: 1014 ± 3 mbar (mean ± SD). For all visits, participants abstained from unaccustomed and high-intensity exercise for 48 h, did not consume alcohol for 24 h and were not allowed to consume caffeine-containing products on the day of testing.

### First visit – familiarization and pre-testing

During the first visit, the main aim was familiarization of the participants with all testing procedures. Height was measured (Nagata S/N CH2882, Taiwan). Body composition was measured from a full body scan using dual X-ray absorptiometry (Lunar iDXA, enCORE software V18, GE Healthcare, Madison, WI, USA). This was followed by a 2 × 10-min measurement in rest in a supine position of cardiovascular responses (Physioflow^®^, Bristol, PA, USA) with the participant fitted with skin electrodes (Ambu^®^, Bluesensor, Malaysia) according to manufacturer guidelines. Recording with a portable breath-by-breath system (Cortex Metalyzer 3B, CORTEX Biophysik GmbH, Leipzig, Germany) allowed simultaneous measurements of the respiratory and metabolic responses. The measurements in rest were taken with the lights dimmed, no background music and no use of electronic devices with a 5 min break between the 2 × 10 min measurements. The lowest recording for oxygen consumption during the 2 × 10 min measurements was taken as the one metabolic equivalent of task (1-MET), i.e. 3.31 ± 0.66 mL·kg^− 1^·min^− 1^ (mean ± SD). The individual 1-MET value was used for the calculation of the treadmill speed (see below) for the walking-induced 5-MET intensity (i.e. moderate-intensity exercise) with the incremental walking test. The incremental walking test was initiated with a starting speed of 2 km·h^− 1^ with a stage increment of 1 km·h^− 1^ (Marquette Series 2000 treadmill, USA). Each stage lasted 8-min and normally 4 to 5 stages were completed in order to achieve an oxygen consumption in the last stage close or over the 5-MET value based on the Cortex feedback during the test. Precise calculation of the oxygen consumption in each stage was based on the recordings of the expired air in the last 3 min of each stage. The linear relationship between the treadmill speed and oxygen consumption (Arts and Kuipers 1994) expressed as the metabolic equivalent (r^2^ = 0.9406 ± 0.0346, mean ± SD) allowed the calculation of the walking speed at 5-METs (i.e. moderate intensity exercise).

### Dosing strategy and experimental visits

Before the experimental visits for the placebo and NZBC extract condition, the dosing strategy involved a daily dose for 14 days. The optimal dosing strategy for optimal responses of anthocyanin-rich extract is not known. However, a 14-day intake duration with a dose of 210 mg of blackcurrant anthocyanins daily was chosen due to the absence of metabolic responses in a study with 7-day intake and similar dose in Southeast Asian males (Willems et al. 2018). The female participants consumed two doses of capsulated placebo (each capsule containing 300 mg microcrystalline cellulose M102) or anthocyanin-rich NZBC extract (CurraNZ, Health Currancy Ltd, Surrey, United Kingdom). According to company information, each capsule of 300 mg with NZBC extract contained 105 mg of anthocyanins, i.e. 35–50% delphinidin-3-rutinoside, 5–20% delphinidin-3-glucoside, 30–45% cyanidin-3-rutinoside, 3–10% cyanidin-3 glucoside. The remaining content of the NZBC extract capsules consisted mainly of natural plant sugars. Each capsule of NZBC extract equals about 85 New Zealand grown blackcurrants. Placebo and NZBC extract capsules were of similar size and identical looking. For the first 13 days, the participants were advised to consume the two capsules daily with breakfast. The final two capsules in each condition were taken 90 min before arrival in the laboratory on the day of testing. Participants were advised to consume the final two capsules with a slice of bread and water. The dosing strategy adopted a washout period that corresponded with the duration of the menstrual cycle. For the cohort, the number of wash-out days was 29 ± 2 days except for one participant which was tested with 56 days in between the placebo and NZBC extract conditions. Capsule compliance was monitored by capsule counts and participant self-report, indicating good adherence to the supplementation protocol. Blinding success was assessed at study completion by asking participants to guess the condition received, with no systematic indication of unblinding. With this dosing strategy in the present study, each female participant was tested in a similar phase of the menstrual cycle (Olenick et al. 2024). The phase of the menstrual cycle was not verified and use of contraceptives was not recorded. Menstrual cycle phase was controlled by scheduling the placebo and NZBC extract conditions within the same self-reported phase of the menstrual cycle for each participant. Information on menstrual cycle timing was obtained by participant self-report. The participants recorded for a period of 48 h their dietary intake before the first experimental visit in either the placebo or NZBC condition and were instructed to replicate the intake for the 48 h for the subsequent visit. The food diaries were analysed with INMUcal v.3 NB.3, with values for nutrition for the Thai food (Institute of Nutrition, Mahidol University, Thailand) to quantify carbohydrate, fat and protein intake and total energy intake (kJ) (Table 1). There were no differences for carbohydrate, fat and protein intake and total energy intake in the placebo and NZBC extract condition (*P* > 0.05).

**Table 1:** Daily dietary intake with absolute values and values relative to body mass

Parameter	Placebo	NZBC extract
Carbohydrate (g)	148 ± 52	136 ± 64
(g∙kg body mass^− 1^)	2.6 ± 0.8	2.4 ± 1.2
Fat (g)	59 ± 26	74 ± 45
(g∙kg body mass^− 1^)	1.0 ± 0.5	1.3 ± 0.9
Protein (g)	71 ± 34	70 ± 24
(g∙kg body mass^− 1^)	1.2 ± 0.6	1.2 ± 0.4
Total energy intake (kJ)	5869 ± 2201	6244 ± 2451
(kJ·body mass^− 1^)	138 ± 101	109 ± 49

Values are means ± SD for 16 female participants (age: 23 ± 5 years, BMI: 22.9 ± 3.8 kg·m^− 2^)

*NZBC* New Zealand blackcurrant.

For the placebo and NZBC extract condition, cardiovascular responses and expired air were taken during 2 × 10 min measurements for rest in supine position and during the 30-min on the treadmill at individualized walking speed. The second 10 min rest measurements were used for analysis. The individualized walking speeds calculated for the moderate-intensity exercise were 4.84 ± 1.00 km·hr^− 1^ (mean ± SD) (range: 3.36–6.90 km·hr^− 1^). During rest and treadmill walking, beat-by-beat and breath-by-breath recording systems were used as described above.

### Calculations and statistical analysis

The respiratory exchange ratio (i.e. RER) was calculated by the ratio of the volume of carbon dioxide produced and the volume of oxygen uptake. The rates of whole-body fat and carbohydrate oxidation were calculated with Eqs. 1 and 2 for moderate to high-intensity exercise from Jeukendrup and Wallis (2005), based on the stoichiometry of glucose and palmitic acid and the assumption of negligible protein oxidation:

1 $$Fat\ oxidation\:\left( {g \cdot min^{{ - 1}} } \right) = 1.695 \times \:\:\dot{V}O_{2} - 1.701\: \times \:\:\dot{V}CO_{2}$$

2 $$Carbohydrate\ oxidation\:\left( {g \cdot min^{{ - 1}} } \right) = 4.210 \times \dot{V}CO_{2} - 2.962 \times \dot{V}O_{2}$$

Due to the number of parameters, sample size in the present study (*n* = 16) was decided based on the sample size used in previous studies in female and male Caucasian participants reporting on cardiovascular and metabolic responses (*n* = 16 men: Sahin et al. 2021, 2023, *n* = 12 women: Willems et al. 2022). However, a sensitivity analysis indicated that with *n* = 16 in the cross-over design and α = 0.05 (two-sided), the study had 80% power to detect within-subject effects of approximately Cohen’s d ≈ 0.75 (i.e., large effects). Due to an absence of information on factors that may affect the responses to the intake of blackcurrant intake, we did not attempt a covariate analysis. The statistical analyses were completed using GraphPad Prism (v5 for Windows, GraphPad Software, La Jolla, USA). Data normality assumptions were tested with the Kolmogorov-Smirnov test with cardiac output in the NZBC condition during rest, systemic vascular resistance in the placebo condition during rest, and stroke volume during exercise in both conditions not passing normality. A Wilcoxon signed ranked test was used for not normally distributed parameters, otherwise two-tailed paired samples t-test. For the individual responses to the intake of NZBC, the smallest worthwhile change (SWC)-based threshold (i.e., 0.2 × SD of the placebo data) was used to assess meaningful individual responses (Swinton et al. 2018) for parameters with a change or trend for change. Individual responses were determined by counts of the differences in the responses between the New Zealand blackcurrant extract and placebo condition beyond the SWC. Effect sizes, i.e. Cohen’s *d* were calculated for changes that were significant or showed a trend for significance, with an effect size of < 0.2 as a trivial, 0.2–0.39 as a small, 0.4–0.69 as a moderate and ≥ 0.7 as a large magnitude of change. Pearson correlation coefficients were calculated for (1) the relationship between fat oxidation at rest and fat oxidation during the moderate-intensity 30-min walk for placebo and NZBC extract conditions, and (2) the relationship between body fat (%) and the change in walking-induced fat oxidation by intake of NZBC extract. Correlation analyses were exploratory and intended to describe associations within conditions. Statistical significance was accepted at *P* < 0.05. Interpretation of 0.05 > *P* ≤ 0.1 (a trend) was according to guidelines by Curran-Everett and Benos (2004). Data are presented as mean ± SD.

## Results

### Physiological and metabolic responses during rest in supine position

In Table 2 are presented the absolute values (mean ± SD) for the physiological and metabolic parameters during rest in a supine position for the placebo and New Zealand blackcurrant extract condition. In rest, the New Zealand blackcurrant extract had no effect on breathing frequency (placebo: 95%CI [14, 17 bpm], NZBC extract: 95%CI [14, 17 bpm]), tidal volume (placebo: 95%CI [345, 415 mL]; NZBC extract: 95%CI [347, 408 mL]), minute ventilation (placebo: 95%CI [5.19, 5.96 L·min^− 1^], NZBC extract: 95%CI [5.32, 6.11 L·min^− 1^]), oxygen uptake in absolute (placebo: 95%CI [174, 196 mL·min^− 1^], NZBC extract: 95%CI [178, 206 mL·min^− 1^]) and relative values (placebo: 95%CI [2.98, 3.49 mL·kg^− 1^·min^− 1^], NZBC extract: 95%CI [2.98, 3.78 mL·kg^− 1^·min^− 1^]), carbon dioxide production (placebo: 95%CI [150, 165 mL·min^− 1^], NZBC extract: 95% CI [150, 173 mL·min^− 1^]), carbohydrate oxidation (placebo: 95%CI [0.106, 0.138 g·min^− 1^], NZBC extract: 95%CI [0.101, 0.137 g·min^− 1^]), fat oxidation (placebo: 95%CI [0.038, 0.055 g·min^− 1^], NZBC extract: 95%CI [0.043, 0.058 g·min^− 1^]) and respiratory exchange ratio (placebo: 95%CI [0.83, 0.88], NZBC extract: 95%CI [0.82, 0.86]). The dosing of 210 mg of blackcurrant anthocyanins for two weeks in Southeast Asian women does not seem to alter cell functions that may result in physiological and metabolic responses during supine rest.

**Table 2:** Physiological and metabolic responses during rest in supine position

Parameter	Placebo	NZBC extract	*p*-value
Breathing frequency (bpm)	15 ± 2	16 ± 2	0.685
Tidal volume (mL)	380 ± 65	377 ± 57	0.896
Minute ventilation (L·min^− 1^)	5.58 ± 0.72	5.72 ± 0.74	0.618
Oxygen consumption (mL·min^− 1^)	185 ± 21	192 ± 26	0.348
Oxygen consumption (mL·kg^− 1^·min^− 1^)	3.24 ± 0.48	3.38 ± 0.75	0.328
Carbon dioxide production (mL·min^− 1^)	157 ± 15	161 ± 22	0.572
Carbohydrate oxidation (g·min^− 1^)	0.122 ± 0.030	0.119 ± 0.034	0.801
Fat oxidation (g·min^− 1^)	0.046 ± 0.016	0.051 ± 0.014	0.182
Respiratory exchange ratio	0.85 ± 0.04	0.84 ± 0.04	0.384

Values are mean ± SD (*n* = 16 Southeast Asian women, age: 23 ± 5 years, BMI: 22.9 ± 3.8 kg·m^− 2^).

Dosing with NZBC extract was two weeks with daily 210 mg of blackcurrant anthocyanins

*NZBC* New Zealand blackcurrant extract,* bpm* breaths per minute

### Cardiovascular responses during rest in a supine position

In Table 3 are presented the absolute values (mean ± SD) for the cardiovascular parameters during rest in a supine position for the placebo and New Zealand blackcurrant extract condition. In rest, New Zealand blackcurrant had no effect on heart rate (placebo: 95%CI [58, 67 beats·min^− 1^]), NZBC extract: 95%CI [56, 64 beats·min^− 1^]), stroke volume (placebo: 95%CI [72, 84 mL]), NZBC extract: 95%CI [73, 83 mL]), cardiac output (placebo: 95%CI [4.47, 5.22 L·min^− 1^]), NZBC extract: 95%CI [4.32, 5.03 L·min^− 1^]) and systemic vascular resistance (placebo: 95%CI [1201, 1481 dynes·s·cm^− 5^]), NZBC extract: 95%CI [1197, 1424 dynes·s·cm^− 5^]). An absence of an effect on the cardiovascular responses by intake of two weeks of 210 mg of blackcurrant anthocyanins indicates that endothelial cell function was not changed.

**Table 3:** Cardiovascular responses during rest in supine position

Parameter	Placebo	NZBC extract	*p*-value
Heart rate (beats·min^− 1^)	63 ± 9	60 ± 8	0.156
Stroke volume (mL)	78 ± 12	78 ± 9	0.962
Cardiac output (L·min^− 1^)	4.84 ± 0.71	4.66 ± 0.66	0.171
Systemic vascular resistance (dynes⋅s⋅cm⁻⁵)	1341 ± 263	1310 ± 213	0.856

Values are mean ± SD (*n* = 16 Southeast Asian women, age: 23 ± 5 years, BMI: 22.9 ± 3.8 kg·m^− 2^).

Dosing with NZBC extract was two weeks with daily 210 mg of blackcurrant anthocyanins

*NZBC* New Zealand blackcurrant extract

### Physiological and metabolic responses during the 30-min treadmill walk at moderate intensity

The aim of the measurements during rest and during an incremental walking test in the first visit was to calculate the treadmill walking speed at a moderate intensity of 5-MET. For the placebo and NZBC extract condition, the responses for oxygen consumption at rest (see methods) and during the 30-min walk showed that the moderate intensity in METs was not different between conditions (placebo: 4.54 ± 0.82 MET; NZBC extract: 4.46 ± 0.87 MET, *P* = 0.577). In Table 4 are presented the physiological and metabolic responses (mean ± SD) during the moderate-intensity treadmill walk for the placebo and New Zealand blackcurrant extract condition. During moderate-intensity treadmill walking, the New Zealand blackcurrant extract had no effect on breathing frequency (placebo: 95%CI [26, 32 bpm], NZBC extract: 95%CI [26, 33 bpm]), tidal volume (placebo: 95%CI [798, 931 mL]; NZBC extract: 95%CI [783, 941 mL]), minute ventilation (placebo: 95%CI [21.30, 27.29 L·min^− 1^], NZBC extract: 95%CI [21.64, 27.96 L·min^− 1^]), oxygen uptake in absolute (placebo: 95%CI [739, 916 mL·min^− 1^], NZBC extract: 95%CI [754, 922 mL·min^− 1^]) and relative values (placebo: 95%CI [12.52, 16.68 mL·kg^− 1^·min^− 1^], NZBC extract: 95%CI [12.67, 17.01 mL·kg^− 1^·min^− 1^]), carbon dioxide production (placebo: 95%CI [643, 802 mL·min^− 1^], NZBC extract: 95% CI [660, 808 mL·min^− 1^]), carbohydrate oxidation (placebo: 95%CI [0.528, 0.734 g·min^− 1^], NZBC extract: 95%CI [0.546, 0.755 g·min^− 1^]), fat oxidation (placebo: 95%CI [0.141, 210 g·min^− 1^], NZBC extract: 95%CI [0.135, 0.212 g·min^− 1^]) and respiratory exchange ratio (placebo: 95%CI [0.85, 0.89], NZBC extract: 95%CI [0.85, 0.90]). The dosing of 210 mg of blackcurrant anthocyanins for two weeks in Southeast Asian women does not seem to alter cell functions that may result in physiological and metabolic responses during moderate-intensity treadmill walking.

**Table 4:** Physiological and metabolic responses during 30-min of moderate intensity treadmill walking

Parameter	Placebo	NZBC extract	*p*-value
Breathing frequency (bpm)	29 ± 6	30 ± 6	0.302
Tidal volume (mL)	865 ± 125	862 ± 148	0.877
Minute ventilation (L·min^− 1^)	24.30 ± 5.62	24.80 ± 5.93	0.330
Oxygen consumption (mL·min^− 1^)	828 ± 167	838 ± 158	0.577
Oxygen consumption (mL·kg^− 1^·min^− 1^)	14.60 ± 3.91	14.84 ± 4.07	0.448
Carbon dioxide production (mL·min^− 1^)	723 ± 149	734 ± 139	0.479
Carbohydrate oxidation (g·min^− 1^)	0.631 ± 0.194	0.651 ± 0.196	0.640
Fat oxidation (g·min^− 1^)	0.175 ± 0.065	0.173 ± 0.073	0.904
Respiratory exchange ratio	0.87 ± 0.04	0.88 ± 0.04	0.734

Values are mean ± SD (*n* = 16 Southeast Asian women, age: 23 ± 5 years, BMI: 22.9 ± 3.8 kg·m^− 2^). Dosing with NZBC extract was two weeks with daily 210 mg of blackcurrant anthocyanins

*NZBC* New Zealand blackcurrant extract. bpm, breaths per minute

**Fig. 1:**
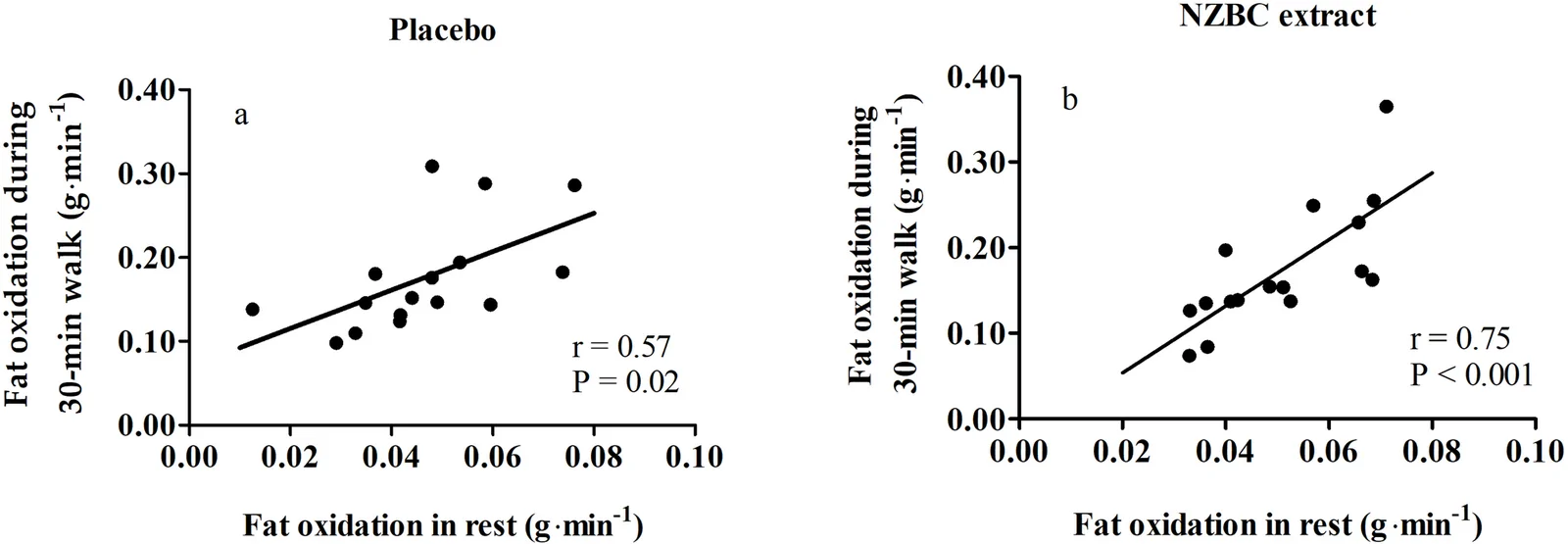
Relationship between whole-body fat oxidation in rest and during a moderate-intensity treadmill walk for 30 min in placebo **a** and New Zealand blackcurrant (NZBC) extract conditions **b**. The pearson correlation coefficient was significant in both conditions

**Fig. 2:**
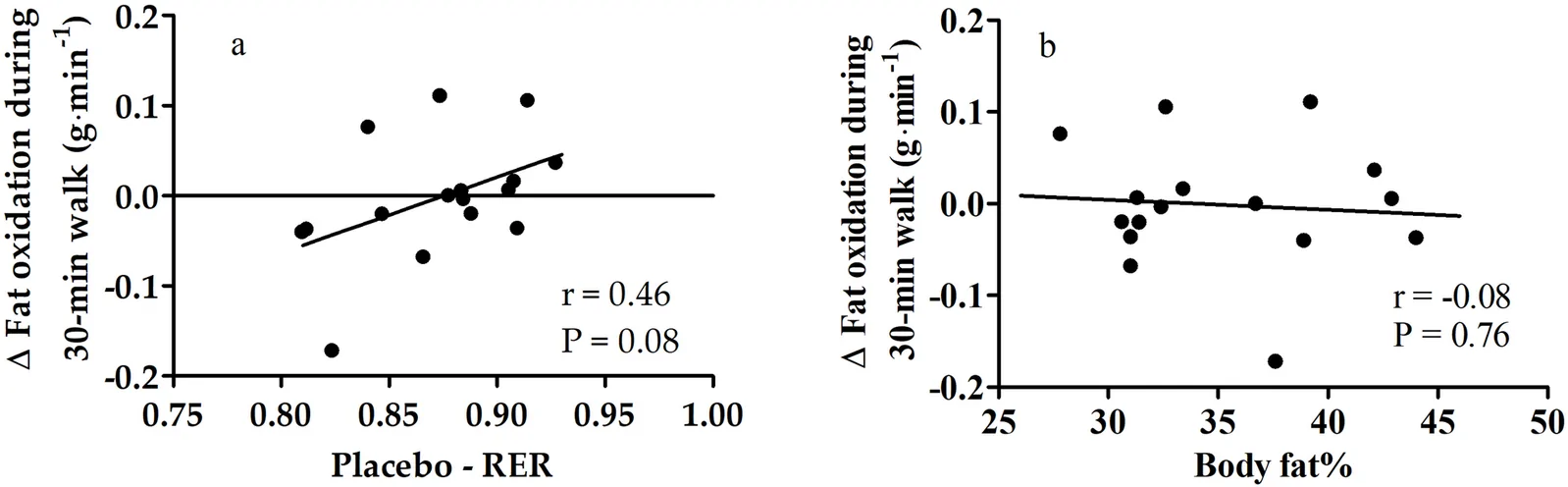
**a** Relationship between walking induced respiratory exchange ratio in the placebo condition and NZBC extract induced change in fat oxidation during the moderate intensity walk; **b** Relationship between body fat (%) and NZBC extract induced change in fat oxidation during the moderate intensity walk. There was a trend for the pearson correlation coefficient to be significant in ** a**

For Southeast Asian women, whole-body fat oxidation in rest and during the moderate-intensity 30-min treadmill walk were significantly correlated for both the placebo (Fig. 1a) and NZBC extract conditions (Fig. 1b). In addition, there was a trend for a significant correlation for the relationship between walking induced respiratory exchange ratio in the placebo condition and NZBC extract induced change in fat oxidation during the moderate-intensity treadmill walk (Fig. 2a). There was no significant correlation between body fat (%) and NZBC extract induced change in fat oxidation during the moderate-intensity treadmill walk.

### Cardiovascular responses during the 30-min treadmill walk at moderate intensity

In Table 5 are presented the absolute values (mean ± SD) for the cardiovascular parameters during rest in a supine position for the placebo and New Zealand blackcurrant extract condition. In rest, New Zealand blackcurrant had no effect on heart rate (placebo: 95%CI [107, 131 beats·min^− 1^]), NZBC extract: 95%CI [108, 131 beats·min^− 1^]), stroke volume (placebo: 95%CI [94, 115 mL]), NZBC extract: 95%CI [95, 114 mL]) and cardiac output (placebo: 95%CI [11.18, 13.17 L·min^− 1^]), NZBC extract: 95%CI [11.33, 13.13 L·min^− 1^]). For systemic vascular resistance (placebo: 95%CI [481, 584 dynes·s·cm^− 5^]), NZBC extract: 95%CI [457, 544 dynes·s·cm^− 5^]), there was a trend for lower values by 5% for the group in the NZBC extract condition with a moderate effect size (*d* = 0.62). Eight of the participants had a change larger than the smallest worthwhile change with for those participants lower values for systemic vascular resistance by 15%. An absence of a clear effect on all cardiovascular responses, but lower systemic vascular resistance in 50% of the participants, indicating whole-body vasodilation by intake of two weeks of 210 mg of blackcurrant anthocyanins during moderate intensity treadmill walking indicates that endothelial cell function was changed in some of the participants.

**Table 5:** Cardiovascular responses during the 30-min of moderate intensity treadmill walking

Parameter	Placebo	NZBC extract	*p*-value
Heart rate (beats·min^− 1^)	119 ± 23	120 ± 22	0.760
Stroke volume (mL)	104 ± 19	104 ± 18	0.737
Cardiac output (L·min^− 1^)	12.17 ± 1.87	12.23 ± 1.69	0.889
Systemic vascular resistance (dynes⋅s⋅cm⁻⁵)	533 ± 97	500 ± 82	0.081

Values are mean ± SD (*n* = 16 Southeast Asian women, age: 23 ± 5 years, BMI: 22.9 ± 3.8 kg·m^− 2^)

Dosing with NZBC extract was two weeks with daily 210 mg of blackcurrant anthocyanins

*NZBC* New Zealand blackcurrant extract.

## Discussion

This is the first study that examines in Southeast Asian women the effect of an anthocyanin-rich dietary supplement on the cardiovascular, physiological and metabolic responses during rest and moderate-intensity exercise. Given the underrepresentation of female and non-Caucasian participants in sports and exercise nutrition research, our findings provide insight into potential ethnicity- and sex-specific differences in responsiveness to anthocyanin supplementation. In the present study, Southeast Asian women (age: 23 ± 5 years, BMI: 22.9 ± 3.8 kg·m^− 2^) did respond to a 2-week daily intake of 210 mg of anthocyanins of an extract made from New Zealand grown blackcurrants with a trend for reduced walking-induced systemic vascular resistance. However, the effect of lower systemic vascular resistance was observed only in 50% of the participants and represented an isolated cardiovascular response. During supine rest and walking-induced responses at moderate intensity (~ 4.5 METs), other cardiovascular (i.e., heart rate, stroke volume, cardiac output), the physiological (i.e., breathing frequency, tidal volume, minute ventilation, oxygen uptake and carbon dioxide production) and metabolic responses (i.e., fat oxidation, carbohydrate oxidation and respiratory exchange ratio) were not altered. The potential effect on systemic vascular resistance needs to be treated with caution as our statistical analysis did not adjust for multiplicity testing and we had for this outcome overlapping confidence intervals. In addition, It needs to be acknowledged that the standard deviations for repeated measurements of systemic vascular resistance and other parameters during rest and moderate-intensity walking in our study were not known.

In White (i.e. Caucasian) women, a dosing strategy with 7-days intake and 210 mg anthocyanins during moderate-intensity treadmill walking (i.e. 4.7 ± 0.4 METs) enhanced walking-induced fat oxidation by 25% and provided lower values for carbohydrate oxidation by 10.8% and for respiratory exchange ratio by 3% (Willems et al. 2022). In the study by Willems et al. (2022), many of the physiological and metabolic responses during moderate-intensity treadmill walking in the placebo condition were similar in comparison with the observations in the present study: e.g. heart rate placebo: 121 ± 17 beats·min^− 1^, minute ventilation placebo: 25.8 ± 7.4 L·min^− 1^, oxygen uptake placebo: 16.1 ± 2.1 mL·kg^− 1^·min^− 1^, and respiratory exchange ratio placebo: 0.86 ± 0.05). A notable difference was that the Southeast Asian women had lower walking-induced fat oxidation than Caucasian women by ~ 27% (Willems et al. (2022): 0.241 ± 0.086, the present study: 0.175 ± 0.065 g·min^− 1^). This is notable because lower exercise-induced fat oxidation capacity has been associated with a greater risk for insulin resistance and metabolic disorders, which are highly prevalent in Asian populations (Wulan et al. 2021). A previous study has also reported lower fat oxidation during exercise in South Asian compared with White European men (Hall et al. 2010). Nevertheless, the alteration of substrate utilization by intake of blackcurrant anthocyanins has been confirmed in other studies as well with Caucasian female and male participants (Strauss et al. 2018; Sahin et al. 2021). The longer intake duration in the present study, albeit without outcome on substrate utilization, was based on supplementation methodology in the study by Willems et al. (2018) in Southeast Asian men in which a 7-day intake with 210 mg of blackcurrant anthocyanins did not provide alterations in substrate utilization in rest and during moderate-intensity treadmill walking. However, the observations by Willems et al. (2018) and the present study confirm that in Southeast Asian women and men, the fat oxidation in rest is associated with exercise-induced fat oxidation and confirming observations in Caucasians by Robinson et al. (2016). In addition, a recent secondary analysis of studies that reported enhanced exercise-induced fat oxidation by intake of anthocyanin-rich blackcurrant showed that the respiratory exchange ratio during exercise in the placebo condition correlated with the change in fat oxidation during the exercise (Willems and Cook 2025). This relationship was confirmed in the present study. However, Fig. 2a suggests considerable inter-individual variability, with some participants showing increased fat oxidation and others showing the opposite, resulting in no overall group effect. This highlights the need for future work to investigate whether specific phenotypes or metabolic profiles determine responsiveness to anthocyanin supplementation. It needs to be noted here as well that even in studies with Caucasians in which there is a group effect for altered substrate utilization with intake of blackcurrant, the optimal dosing strategy with blackcurrant anthocyanins to alter the metabolic responses during rest and exercise is not known. In the present study, the absence of a metabolic response to the intake of anthocyanin-rich blackcurrant extract may suggest in Southeast Asian women that the causal mechanisms for altered substrate utilization were not triggered with the selected dosing strategy and is likely associated with the metabolism of the blackcurrant anthocyanins. The present findings should be interpreted within the context of the study population. As no Caucasian comparator group was included, conclusions are limited to Southeast Asian women, and direct comparisons across ethnic groups cannot be made. The references to previous studies in Caucasian populations were intended to provide contextual background rather than evidence of ethnic differences. Therefore, the observed responses reflect physiological and metabolic characteristics specific to Southeast Asian women under the present experimental conditions. In addition, we did not control for having the women tested in a similar phase of the menstrual cycle. In addition to potential ethnic differences in anthocyanin metabolism and bioavailability, race-specific differences in fat distribution may also have contributed to the observed metabolic responses. It is well established that Asian populations tend to accumulate a greater proportion of visceral adipose tissue relative to subcutaneous fat at a given body mass index or total body fat percentage compared with Caucasian populations, a pattern associated with increased metabolic risk and altered substrate utilization. Visceral adiposity is closely associated with insulin resistance, altered lipid metabolism, and reduced metabolic flexibility, which may influence substrate utilization during rest and exercise. Although total body fat percentage was assessed in the present study, visceral and subcutaneous fat depots were not distinguished in the study design or analysis. Therefore, the potential role of fat distribution influencing responsiveness to anthocyanin-rich New Zealand blackcurrant supplementation could not be evaluated. This represents a limitation of the present study and should be considered when interpreting ethnic differences in metabolic outcomes. Future studies should incorporate direct assessments of fat distribution to better elucidate the interaction between ethnicity, fat distribution, and metabolic responses to anthocyanin supplementation.

Anthocyanin metabolism is a process involving enzymes in the gut, intestines and liver. It is possible that the absence of a metabolic effect to the intake of blackcurrant anthocyanins in Southeast Asians compared to Caucasians may be due to differences in anthocyanin metabolism at least with the selected dosing strategy. For example, it is known that there are differences in the drug-metabolizing enzyme CYP1A2 in Southeast Asian and European individuals due to diet, lifestyle and genetic factors (Perera et al. 2012). CYP1A2 is just one of the many enzymes involved in caffeine metabolism and a recent review by Low et al. (2024) highlights ethnic differences due to genetic variability in these enzymes. As it is not clear which specific enzymes are involved for the handling of blackcurrant anthocyanins, ethnic difference in those enzymes and activity is speculative but cannot be excluded. In addition, differences in the gut microbiome due to ethnicity and diet by Southeast Asian and Caucasian participants in our studies on blackcurrant effects is very likely (see Gupta et al. 2017) and affecting anthocyanin metabolism (Baky et al. 2022). It is also possible that differences in gut microbiome exist due to the Southeast Asian participants being heat-acclimatised, albeit as far as we know, it has only been shown in rats that exposure to 35 ± 1 °C, 60 ± 5% humidity and for 120 min per day for 28 days changes the gut microbiome (Cao et al. 2022). We did not measure plasma or urinary anthocyanin metabolites, which is a limitation of the present study. Without bioavailability data, it is not possible to determine whether the absence of metabolic effects reflects poor absorption, rapid clearance, or ethnic-specific differences in metabolism with the selected dosing strategy. The role of genes, ethnicity, age, environmental conditions, diet and other lifestyle factors need to be understood how they can affect the plasma bioavailability of anthocyanins and anthocyanin-induced metabolites and whether they can alter whole-body metabolism of the macronutrients fats and carbohydrates. The reader is referred to reviews by Lila et al. (2016) and Eker et al. (2019) for the handling of anthocyanins and factors that contribute to the bioavailability of anthocyanins and anthocyanin-induced metabolites. Thus, future studies should combine supplementation trials with metabolite profiling and microbiome analysis to clarify mechanisms underlying inter-individual and ethnic differences in anthocyanin responsiveness.

In the present study, the intake of NZBC extract showed a trend for lower walking-induced systemic vascular resistance. Lower exercise-induced systemic vascular resistance with intake of anthocyanin-rich blackcurrant was also shown in Southeast Asian men (Willems et al. 2018). This would suggest that Southeast Asians can have beneficial effects on exercise-induced endothelial function with intake of an anthocyanin-rich supplement. The purported effects of anthocyanin-rich blackcurrant during exercise may be due to the presence and ability of some anthocyanin-induced metabolites to reduce oxidative stress. Based on many studies with Caucasian and Asian participants, cardiovascular responses are possible with intake of blackcurrant anthocyanins. In Japanese older adults (age: 73.3 ± 1.7 years), intake of anthocyanin-rich blackcurrant reduced central arterial stiffness and blood pressure (Okamoto et al. 2020). In addition, in Caucasian women and men (age: 29 ± 8 years), intake of anthocyanin-rich blackcurrant reduced post-exercise blood pressure (Cook et al. 2025). A study in South Asian men showed that there was reduced flow-mediated vasodilation (Cubbon et al. 2010). We do not know whether walking-induced vasodilation was blunted in Southeast Asian women, but it is encouraging that at least 50% of the participants showed responsiveness to the intake of anthocyanin-rich blackcurrant indicating endothelial function alterations. It is also worth noting that the systemic vascular resistance was lower by 10% during treadmill walking in Southeast Asian men with intake of blackcurrant (placebo: 779 ± 267; NZBC extract: 697 ± 245 dyn·s·cm^− 5^; *p* = 0.048) (Willems et al. 2018). Future work should examine whether a larger response rate for changes in systemic vascular resistance is possible in Southeast Asian women with dosing strategies with longer intake duration or larger doses.

Several limitations should be acknowledged. Menstrual cycle phase and use of contraceptives were not recorded; thus, hormonal variability and potential effects of contraceptive use cannot be excluded, despite scheduling visits within the same phase of the menstrual cycle. The laboratory temperature of 27.5 ± 0.9 °C may also have influenced the variation in cardiovascular and metabolic responses, but conditions were identical across trials. Dietary intake and caffeine consumption were controlled through participant instructions and self-reported records. Still, variability in dietary intake may have contributed to inter-individual differences. Given the modest sample size, stratification or statistical adjustment for body composition was not feasible; although total body fat percentage was measured, visceral and subcutaneous adipose tissue depots were not distinguished, and potential influences of adiposity patterns on responsiveness for systemic vascular resistance cannot be excluded. Finally, bioavailability of anthocyanins and metabolites was not assessed, leaving uncertainty about the relationship between intake and systemic exposure. From a practical perspective, our findings highlight the importance of not generalizing supplementation outcomes from Caucasian to Asian populations. While anthocyanin-rich blackcurrant extract may support vascular responses in some Southeast Asian women, the lack of consistent metabolic benefits suggests that personalized approaches, taking into account ethnicity, sex, and metabolic phenotype, may be required before recommending anthocyanins for performance or health applications.

It is concluded that Southeast Asian women can show responsiveness during moderate-intensity exercise to the intake of anthocyanin-rich blackcurrant with lowering of the systemic vascular resistance indicating enhanced whole-body exercise-induced vasodilation. This beneficial anthocyanin-induced cardiovascular response was not associated with any other physiological, metabolic and cardiovascular responses at rest and during moderate-intensity treadmill walking. Future work is required to optimize dosing strategies for intake of blackcurrant anthocyanins in individuals with different ethnicities and have experimental designs with comparative groups to examine cardiovascular and metabolic responses that can be indicative of health benefits.

## Abbreviations

BMI: body mass index
CO: cardiac output
DBP: diastolic blood pressure
NZBC: New Zealand blackcurrant
PLA: placebo
SBP: systolic blood pressure

## References

[CR2] Akins JD, Curtis BM, Patik JC, Olvera G, Nasirian A, Campbell JC, Shiva S, Brothers RM (2021) Blunted hyperemic response to mental stress in young, non-Hispanic black men is not impacted by acute dietary nitrate supplementation. J Appl Physiol 130(5):1510–1521. 10.1152/japplphysiol.00453.2020 33764167

[CR3] Anders JPV, Neltner TJ, Smith RW, Keller JL, Housh TJ, Daugherty FJ, Tempesta MS, Dash AK, Munt DJ, Schmidt RJ, Johnson GO (2021) The effects of phosphocreatine disodium salts plus blueberry extract supplementation on muscular strength, power, and endurance. J Int Soc Sports Nutr 18(1):60. 10.1186/s12970-021-00456-y 34503541 PMC8427883

[CR4] Arts FJP, Kuipers H (1994) The relation between power output, oxygen uptake and heart rate in male athletes. Int J Sports Med 15(05):228–231 7960315 10.1055/s-2007-1021051

[CR5] Baky MH, Elshahed M, Wessjohann L, Farag MA (2022) Interactions between dietary flavonoids and the gut microbiome: a comprehensive review. Br J Nutr 128(4):577–591. 10.1017/S0007114521003627 34511152

[CR6] Cao Y, Liu Y, Dong Q, Wang T, Niu C (2022) Alterations in the gut microbiome and metabolic profile in rats acclimated to high environmental temperature. Microb Biotechnol 15(1):276–288. 10.1111/1751-7915.13772 33620148 PMC8719808

[CR7] Capó X, Martorell M, Sureda A, Riera J, Drobnic F, Tur JA, Pons A (2016) Effects of almond-and olive oil-based Docosahexaenoic-and vitamin E-enriched beverage dietary supplementation on inflammation associated to exercise and age. Nutrients 8(10):619. 10.3390/nu8100619 27735833 PMC5084007

[CR8] Cheynier V (2005) Polyphenols in foods are more complex than often thought. Am J Clin Nutr 81(1):223. 10.1093/ajcn/81.1.223S. S-229S 15640485

[CR9] Cikiriz N, Milosavljevic I, JakovljevicB, Bolevich S, Jeremic J, Nikolic Turnic T, Mitrovic M, Srejovic I, Bolevich S, Jakovljevic V (2021) The influences of chokeberry extract supplementation on redox status and body composition in handball players during competition phase. Can J Physiol Pharmacol 99(1):42–47. 10.1139/cjpp-2020-0095 32640181

[CR10] Cook M, Myers SD, Blacker SD, Willems MET (2015) New Zealand blackcurrant extract improves cycling performance and fat oxidation in cyclists. Eur J Appl Physiol 115(11):2357–2365. 10.1007/s00421-015-3215-8 26175097

[CR11] Cook MD, Myers SD, Gault ML, Edwards VC, Willems MET (2017) Cardiovascular function during supine rest in endurance-trained males with New Zealand blackcurrant: A dose–response study. Eur J Appl Physiol 117(2):247–254. 10.1007/s00421-016-3512-x 28013387

[CR12] Cook MD, Shan Y, Willems MET (2025) Effects of New Zealand Black Currant Extract on Exercising Substrate Utilization and Postexercise Blood Pressure in Men and Women. Int J Sport Nutr Exerc Metabol 35(2):150–161. 10.1123/ijsnem.2024-0108 39746353

[CR13] Cubbon RM, Murgatroyd SR, Ferguson C, Bowen TS, Rakobowchuk M, Baliga V, Cannon D, Rajwani A, Abbas A, Kahn M, Birch KM (2010) Human exercise-induced circulating progenitor cell mobilization is nitric oxide-dependent and is blunted in South Asian men. Arterioscl Thromb Vasc Biol 30(4):878–884. 10.1161/ATVBAHA.109.201012 20110574

[CR14] Curran-Everett D, Benos DJ (2004) Guidelines for reporting statistics in journals published by the American Physiological Society. Am J Physiol Regul Integr Comp Physiol 287(2):R247–R249. 10.1152/ajpregu.00346.2004 15789454

[CR15] Eker ME, Aaby K, Budic-Leto I, Rimac Brnčić S, El SN, Karakaya S, Simsek S, Manach C, Wiczkowski W, de Pascual-Teresa S (2019) A review of factors affecting anthocyanin bioavailability: Possible implications for the inter-individual variability. Foods 9(1):2. 10.3390/foods9010002 31861362 PMC7023094

[CR16] Garthe I, Maughan RJ (2018) Athletes and supplements: prevalence and perspectives. Int J Sport Nutr Exerc Metabol 28(2):126–138. 10.1123/ijsnem.2017-0429 29580114

[CR17] Gupta VK, Paul S, Dutta C (2017) Geography, ethnicity or subsistence-specific variations in human microbiome composition and diversity. Front Microbiol 8:1162. 10.3389/fmicb.2017.01162 28690602 PMC5481955

[CR18] Hall LM, Moran CN, Milne GR, Wilson J, MacFarlane NG, Forouhi NG, Hariharan N, Salt IP, Sattar N, Gill JM (2010) Fat oxidation, fitness and skeletal muscle expression of oxidative/lipid metabolism genes in South Asians: implications for insulin resistance? PLoS ONE 5(12):e14197. 10.1371/journal.pone.0014197 21152018 PMC2995737

[CR19] He J, Giusti MM (2010) Anthocyanins: natural colorants with health-promoting properties. Annu Rev Food Sci Technol 1(1):163–187. 10.1146/annurev.food.080708.100754 22129334

[CR20] Hlinský T, Kumstát M, Vajda P (2020) Effects of dietary nitrates on time trial performance in athletes with different training status: Systematic review. Nutrients 12(9):2734. 10.3390/nu12092734 32911636 PMC7551808

[CR21] Howatson G, Snaith GC, Kimble R, Cowper G, Keane KM (2022) Improved endurance running performance following haskap berry (Lonicera caerulea L.) ingestion. Nutrients 14(4):780. 10.3390/nu14040780 35215430 PMC8877138

[CR22] Jeukendrup AE, Wallis GA (2005) Measurement of substrate oxidation during exercise by means of gas exchange measurements. Int J Sports Med 26(S1):S28–S37. 10.1055/s-2004-830512 15702454

[CR23] Lila MA, Burton-Freeman B, Grace M, Kalt W (2016) Unraveling anthocyanin bioavailability for human health. Annu Rev Food Sci Technol 7(1):375–393. 10.1146/annurev-food-041715-033346 26772410

[CR24] Low JJL, Tan BJW, Yi LX, Zhou ZD, Tan EK (2024) Genetic susceptibility to caffeine intake and metabolism: a systematic review. J Translational Med 22(1):961. 10.1186/s12967-024-05737-z PMC11515775 39438936

[CR25] Margaritelis NV, Nastos GG, Vasileiadou O, Chatzinikolaou PN, Theodorou AA, Paschalis V, Vrabas IS, Kyparos A, Fatouros IG, Nikolaidis MG (2023) Inter-individual variability in redox and performance responses after antioxidant supplementation: a randomized double blind crossover study. Acta Physiol 238(4):e14017. 10.1111/apha.14017 37401190

[CR26] Okamoto T, Hashimoto Y, Kobayashi R, Nakazato K, Willems MET (2020) Effects of blackcurrant extract on arterial functions in older adults: A randomized, double-blind, placebo-controlled, crossover trial. Clin Exp Hypertens 42(7):640–647. 10.1080/10641963.2020.1764015 32396017

[CR27] Olenick AA, Pearson RC, Jenkins NT (2024) Impact of aerobic fitness status, menstrual cycle phase, and oral contraceptive use on exercise substrate oxidation and metabolic flexibility in females. Appl Physiol Nutr Metabol 49(1):93–104. 10.1139/apnm-2023-0101 37657080

[CR28] Peeling P, Castell LM, Derave W, de Hon O, Burke LM (2019) Sports foods and dietary supplements for optimal function and performance enhancement in track-and-field athletes. Int J Sport Nutr Exerc Metabol 29(2):198–209. 10.1123/ijsnem.2018-0271 30299192

[CR31] Perera V, Gross AS, McLachlan AJ (2012) Influence of environmental and genetic factors on CYP1A2 activity in individuals of South Asian and European ancestry. Clin Pharmacol Ther 92(4):511–519. 10.1038/clpt.2012.139 22948892

[CR29] Perkins IC, Blacker SD, Willems MET (2024) Individual responses to repeated dosing with anthocyanin-rich New Zealand blackcurrant extract during high-intensity intermittent treadmill running in active males. Nutrients 16(24):4253. 10.3390/nu16244253 39770875 PMC11677273

[CR30] Ramos-Romero S, Léniz A, Martínez‐Maqueda D, Amézqueta S, Fernández‐Quintela A, Hereu M, Torres JL Portillo MP Pérez‐Jiménez J 92021) Inter‐individual variability in insulin response after grape pomace supplementation in subjects at high cardiometabolic risk: role of microbiota and miRNA. Mol Nutr Food Res. 10.1002/mnfr.202000113 33202108

[CR32] Robinson SL, Chambers ES, Fletcher G, Wallis GA (2016) Lipolytic markers, insulin and resting fat oxidation are associated with maximal fat oxidation. Int J Sports Med 37(08):607–613. 10.1055/s-0042-100291 27116342

[CR33] Şahin MA, Bilgiç P, Montanari S, Willems MET (2021) Intake duration of anthocyanin-rich New Zealand blackcurrant extract affects metabolic responses during moderate intensity walking exercise in adult males. J Diet Suppl 18(4):406–417. 10.1080/19390211.2020.1783421 32578472

[CR34] Şahin MA, Bilgiç P, Montanari S, Willems MET (2023) Intake duration of anthocyanin-rich New Zealand blackcurrant extract affects cardiovascular responses during moderate-intensity walking but not at rest. J Diet Suppl 20(3):428–443. 10.1080/19390211.2021.2005214 34791970

[CR35] Salem A, Trabelsi K, Jahrami H, Alrasheed MM, Boukhris O, Puce L, Bragazzi NL, Ammar A, Glenn JM, Chtourou H (2024) Branched-chain amino acids supplementation and post-exercise recovery: an overview of systematic reviews. J Am Nutr Assoc 43(4):384–396. 10.1080/27697061.2023.2297899 38241335

[CR36] Šimerdová B, Bobríková M, Lhotská I, Kaplan J, Křenová A, Šatínský D (2021) Evaluation of anthocyanin profiles in various blackcurrant cultivars over a three-year period using a fast HPLC-DAD method. Foods 10(8):1745. 10.3390/foods10081745 34441523 PMC8394496

[CR37] Smith ES, McKay AK, Kuikman M, Ackerman KE, Harris R, Elliott-Sale KJ, Stellingwerff T, Burke LM (2022) Auditing the representation of female versus male athletes in sports science and sports medicine research: evidence-based performance supplements. Nutrients 14(5):953. 10.3390/nu14050953 35267928 PMC8912470

[CR38] Solnier J, Chang C, Pizzorno J (2023) Consideration for flavonoid-containing dietary supplements to tackle deficiency and optimize health. Int J Mol Sci 24(10):8663. 10.3390/ijms24108663 37240008 PMC10218363

[CR39] Strauss JA, Willems MET, Shepherd SO (2018) New Zealand blackcurrant extract enhances fat oxidation during prolonged cycling in endurance-trained females. Eur J Appl Physiol 118(6):1265–1272 29619595 10.1007/s00421-018-3858-3 PMC5966492

[CR40] Swinton PA, Hemingway BS, Saunders B, Gualano B, Dolan E (2018) A statistical framework to interpret individual response to intervention: paving the way for personalized nutrition and exercise prescription. Front Nutr 5:41. 10.3389/fnut.2018.00041 29892599 PMC5985399

[CR41] Wang Y, Wang Y, Shehzad Q, Su Y, Xu L, Yu L, Zeng W, Fang Z, Wu G, Wei W, Jin Q (2024a) Does omega-3 PUFAs supplementation improve metabolic syndrome and related cardiovascular diseases? A systematic review and meta-analysis of randomized controlled trials. Crit Rev Food Sci Nutr 64(26):9455–9482. 10.1080/10408398.2023.2212817 37222574

[CR42] Wang Z, Qiu B, Li R, Han Y, Petersen C, Liu S, Zhang Y, Liu C, Candow DG, Del Coso J (2024b) Effects of creatine supplementation and resistance training on muscle strength gains in adults < 50 years of age: A systematic review and meta-analysis. Nutrients 16(21):3665. 10.3390/nu16213665 39519498 PMC11547435

[CR44] Willems MET, Cook MD (2025) Alterations of exercise-induced carbohydrate and fat oxidation by anthocyanin-rich New Zealand blackcurrant are associated with the pre-intervention metabolic function: a secondary analysis of randomized crossover trials. Nutrients 17(6):997. 10.3390/nu17060997 40290003 PMC11945279

[CR46] Willems MET, Silva JDS, Cook MD, Blacker SD (2017) Beneficial effects on fasting insulin and postprandial responses through 7-day intake of New Zealand blackcurrant powder. FFHD 7(7):483–493. 10.31989/ffhd.v7i7.335

[CR45] Willems MET, Parktin N, Widjaja W, Ajjimaporn A (2018) Effect of New Zealand blackcurrant extract on physiological responses at rest and during brisk walking in southeast asian men: A randomized, double-blind, placebo-controlled, crossover study. Nutrients 10(11):1732. 10.3390/nu10111732 30424482 PMC6266587

[CR43] Willems MET, Banic M, Cadden R, Barnett L (2022) Enhanced walking-induced fat oxidation by New Zealand blackcurrant extract is body composition-dependent in recreationally active adult females. Nutrients 14(7):1475. 10.3390/nu14071475 35406087 PMC9002771

[CR47] World Health Organisation (2004) Expert consultation: Appropriate body-mass index for Asian populations and its implications for policy and intervention strategies. Lancet 363:157–163. 10.1016/S0140-6736(03)15268-3 14726171

[CR48] Wu X, Beecher GR, Holden JM, Haytowitz DB, Gebhardt SE, Prior RL (2006) Concentrations of anthocyanins in common foods in the United States and estimation of normal consumption. J Agric Food Chem 54(11):4069–4075. 10.1021/jf060300l 16719536

[CR49] Wulan SN, Raza Q, Prasmita HS, Martati E, Maligan JM, Mageshwari U, Fatima I, Plasqui G (2021) Energy metabolism in relation to diet and physical activity: A South Asian perspective. Nutrients 13(11): 3776. 10.3390/nu13113776 PMC8617748 34836031

[CR52] Yamaguchi S, Inami T, Nishioka T, Morito A, Ishiyama K, Murayama M (2025) The Effects of Creatine Monohydrate Supplementation on Recovery from Eccentric Exercise-Induced Muscle Damage: A Double-Blind, Randomized, Placebo-Controlled Trial Considering Sex and Age Differences. Nutrients 17(11):1772. 10.3390/nu17111772 40507040 PMC12157024

